# Remodeling of the Actin Network Associated with the Non-Structural Protein 1 (NS1) of West Nile Virus and Formation of NS1-Containing Tunneling Nanotubes

**DOI:** 10.3390/v11100901

**Published:** 2019-09-27

**Authors:** Wilhelm Furnon, Pascal Fender, Marie-Pierre Confort, Sophie Desloire, Sawitree Nangola, Kuntida Kitidee, Caroline Leroux, Maxime Ratinier, Frédérick Arnaud, Sylvie Lecollinet, Pierre Boulanger, Saw-See Hong

**Affiliations:** 1 Université de Lyon, University Claude Bernard Lyon 1, INRA, EPHE, IVPC, UMR754, Viral Infections & Comparative Pathology, Cedex 07, 69366 Lyon, France; wilhelm.furnon@glasgow.ac.uk (W.F.); marie-pierre.confort@univ-lyon1.fr (M.-P.C.); sophie.desloire@univ-lyon1.fr (S.D.); caroline.leroux@univ-lyon1.fr (C.L.); maxime.ratinier@univ-lyon1.fr (M.R.); frederick.arnaud@univ-lyon1.fr (F.A.); pierre.boulanger22@gmail.com (P.B.); saw-see.hong@univ-lyon1.fr (S.-S.H.); 2 Institut de Biologie Structurale, CNRS UMR 5075, 38042 Grenoble, France; pascal.fender@ibs.fr; 3 Department of Medical Technology, School of Allied Health Sciences, University of Phayao, Phayao 56000, Thailand; goy_ahsup2012@hotmail.com; 4 Center for Research & Innovation, Faculty of Medical Technology, Mahidol University, Nakhon Pathom 73170, Thailand; kuntida.kit@mahidol.edu; 5 EPHE, PSL Research University, INRA, Université de Lyon, University Claude Bernard Lyon 1, UMR754, IVPC, Cedex 07, 69366 Lyon, France; 6 UMR-1161 Virology, ANSES, INRA, Ecole Nationale Vétérinaire d’Alfort, ANSES Animal Health Laboratory, EURL on Equine Diseases, 94704 Maisons-Alfort, France; sylvie.lecollinet@anses.fr; 7 Institut National de la Santé et de la Recherche Médicale, 101, rue de Tolbiac, Cedex 13, 75654 Paris, France

**Keywords:** flavivirus, West Nile virus, non-structural protein 1 (NS1), cytoskeleton, actin network, tunneling nanotubes

## Abstract

The cellular response to the recombinant NS1 protein of West Nile virus (NS1^WNV^) was studied using three different cell types: Vero E6 simian epithelial cells, SH-SY5Y human neuroblastoma cells, and U-87MG human astrocytoma cells. Cells were exposed to two different forms of NS1^WNV^: (i) the exogenous secreted form, sNS1^WNV^, added to the extracellular milieu; and (ii) the endogenous NS1^WNV^, the intracellular form expressed in plasmid-transfected cells. The cell attachment and uptake of sNS1^WNV^ varied with the cell type and were only detectable in Vero E6 and SH-SY5Y cells. Addition of sNS1^WNV^ to the cell culture medium resulted in significant remodeling of the actin filament network in Vero E6 cells. This effect was not observed in SH-SY5Y and U-87MG cells, implying that the cellular uptake of sNS1^WNV^ and actin network remodeling were dependent on cell type. In the three cell types, NS1^WNV^-expressing cells formed filamentous projections reminiscent of tunneling nanotubes (TNTs). These TNT-like projections were found to contain actin and NS1^WNV^ proteins. Interestingly, similar actin-rich, TNT-like filaments containing NS1^WNV^ and the viral envelope glycoprotein E^WNV^ were also observed in WNV-infected Vero E6 cells.

## 1. Introduction

Flaviviruses, belonging to the *Flaviviridae* family, are enveloped, positive-strand RNA viruses that can be transmitted to humans by mosquito and tick bites. Flaviviruses such as dengue virus, Zika virus, West Nile virus (WNV), Japanese encephalitis, and yellow fever virus are human pathogens that cause diseases varying from asymptomatic infections or febrile illness to encephalitis, meningitis, or hemorrhagic shock, all of which can have a possible fatal outcome [1]. The genomes of flaviviruses encode a single viral polyprotein that is processed by viral and host cell proteases to give three structural proteins, namely, C (core), prM/M (membrane), and E (envelope); and seven non-structural proteins, namely, NS1, NS2A, NS2B, NS3, NS4A, NS4B, and NS5 [2].

The non-structural protein NS1 has a molecular weight of 46 to 55 kDa, depending on its N-glycosylation status. NS1 is synthesized as a monomer, which dimerizes after post-translational modification in the lumen of the rough endoplasmic reticulum [3], and is secreted into the extracellular space as a hexameric lipoprotein particle [1,4]. During flavivirus infections, the NS1 protein exists in multiple oligomeric forms, and is found either intracellularly and extracellularly [5,6,7]. Three different forms of NS1 have been described: an intracellular membrane-associated form [8,9], a cell surface-bound form, and a secreted form (sNS1) [4,10,11,12]. The intracellular dimeric NS1 colocalizes with dsRNA and other components of the viral replication complex, and plays an essential cofactor role in virus replication [1,13]. NS1 is not present in the viral particles, but is found as membrane-associated dimers and secreted, lipid-associated hexamers [1,4].

In recent years, there has been renewed interest in the role of the NS1 protein in viral pathogenesis. The NS1 genes of flaviviruses share a high degree of sequence homology, and crystallographic analyses of NS1 crystals have shown that their three-dimensional (3D) structures are almost identical [10]. Numerous studies have demonstrated the multifunctional nature of NS1. Intravenous administration of mice with the dengue virus (DENV) NS1 secreted form (sNS1^DENV^) showed accumulation of sNS1^DENV^ in the liver and its association with hepatocytes [14]. Further, sNS1^DENV^ can bind directly to the plasma membrane of uninfected epithelial and fibroblastic cells in vitro via interactions with glycosaminoglycans (heparan sulfate or chondroitin sulfate E) or Toll-like receptors (TLRs) [10,15,16,17,18]. Interestingly, sNS1^DENV^ has differential cell-binding specificity, as it binds efficiently to epithelial and mesenchymal cells but poorly to peripheral blood cells.

In the extracellular milieu, sNS1 exerts a positive effect on flavivirus infection and pathogenesis through its interaction with multiple components of the innate and adaptive immune systems, and its implication in the viral evasion from the host antiviral response [1,10,19,20,21,22]. NS1 also inhibits the host interferon-β production by acting as an antagonist of the RIG-I-like-receptor (RLR)-mediated pathway [23]. Blood-circulating and cell-surface-associated sNS1 are both highly immunogenic, and sNS1 protein or anti-NS1 antibodies are early diagnostic biomarkers of flavivirus infection used in clinical assays [1,9,10,16,24,25,26,27].

As with many other viruses, flaviviruses subvert and utilize the cytoskeleton to infect their host cells [28,29]. This has been well-documented by cytological analyses in the early steps of virus internalization and intracellular trafficking, and also in the late steps of viral particle assembly and release [28,30,31,32,33,34,35,36,37]. Direct evidence of NS1–actin interaction was provided by a study showing that NS1^DENV^ protein was recovered from the cytoskeletal fraction of DENV-infected human endothelial cells (EA.hy926) at relatively late times (ca. 12 h) post infection [35]. More recently, the β-actin-related protein T1 was identified in the interactome of NS1^DENV^ with different human cell types, namely, Raji (lymphoblastoid), HeLa (epithelial), and HAP1 (myeloid) cells [38], and with the actin-related protein 10 among the interactors of NS1^DENV^ in the human hepatocellular carcinoma cell HepG2 [39].

Tunneling nanotubes (TNTs) are actin-rich projections that facilitate long-distance intercellular communication [40,41,42,43]. They are thin membrane channels that form intercellular bridges and allow direct, cell-to-cell transfer of organelles and cytoplasmic molecules [44]. The architecture and ultrastructural organization of neuronal TNTs were recently elucidated by using correlative focus ion beam-scanning and transmission electron microscopy (EM) in combination with cryo-fluorescence microscopy, cryo-EM, and cryo-electron tomography [45]. Recent studies have shown that viruses can take advantage of these channels for their intercellular transmission, with minimal exposure to the extracellular environment [46]. This has been described for retroviruses [47,48,49,50,51,52], influenza virus [53], vaccinia virus [54,55], pseudorabies virus [56,57], and herpes simplex virus [58], as well as for neuron-to-neuron transfer of pathological Tau protein assemblies and prion-like proteins in neurodegenerative diseases [59,60,61].

In this study, we further investigated the multiple properties of the NS1 protein of the West Nile virus (NS1^WNV^). We tested the capacity of the extracellular, secreted form of recombinant NS1^WNV^ (sNS1^WNV^) to attach and enter cells, and to possibly affect the cytoskeletal network upon its cellular uptake. The cellular alterations associated with the expression of recombinant NS1^WNV^ proteins were also analyzed. Three different cell types were used in our study: simian epithelial cells (Vero E6), human neuronal cells (SH-SY5Y), and human glial cells (U-87MG). We observed that sNS1^WNV^ attachment to cells and cellular uptake varied with the cell type and was more efficient in Vero E6 and SH-SY5Y cells than in U-87MG. Modification of the cellular actin filament network was found to be associated with NS1^WNV^ in different types of experimental setups: (i) Addition of purified secreted sNS1^WNV^ protein to the cell culture medium (exogenous sNS1^WNV^) resulted in a significant but transient remodeling of the actin cytoskeleton in Vero E6 cells. (ii) Upon intracellular expression of recombinant NS1^WNV^ protein (endogenous NS1^WNV^), actin-rich, TNT-like projections containing NS1^WNV^ were observed in the three cell types. (iii) Similar filamentous projections forming intercellular bridges were observed in WNV-infected Vero E6 cells in the late stages of the virus cycle, and these actin-rich filaments were found to contain NS1^WNV^ and the viral envelope glycoprotein E^WNV^.

Interestingly, incubation of cells with sNS1^WNV^, added before or concomitantly to WNV, did not significantly change the infectiveness of the virus in any of the studied cell types. Collectively, our results confirmed the multiplicity of functions associated with the flaviviral NS1 proteins, but the common molecular mechanism remains to be determined.

## 2. Materials and Methods

### 2.1. Cells and Virus

Human epithelial cell HEK-293T (ATCC^®^ CRL-3216™), simian epithelial cell Vero E6 (ATCC^®^ CRL-1586™), human astrocytoma cell U-87MG (ATCC^®^ HTB-14™), and human neuroblastoma cell SH-SY5Y (ATCC^®^ CRL-2266™) were provided by CelluloNet (SFR-Biosciences, Lyon, France). HEK-293T, Vero E6, and U-87MG cells were grown in Dulbecco’s modified Eagle’s medium (DMEM; GlutaMAX, high glucose, Gibco™, Thermo Fisher Scientific, Carlsbad, CA, USA) supplemented with 10% heat-inactivated fetal bovine serum (FBS; Gibco™). SH-SY5Y cells were grown in DMEM/nutrient mixture F-12 (DMEM/F-12, Ham 1:1, GlutaMAX, Gibco™) supplemented with 2% heat-inactivated FBS. Cell cultures were maintained at 37 °C and 5% CO_2_. For the production of recombinant sNS1 protein, geneticin™-G418 (500 μg/mL, Gibco™) was added to the culture medium of HEK-293T cells 3 days before seeding them for transfection.

The IS-98-ST1 strain of WNV (provided by Dr. S. Lecollinet; ANSES-ENVA, Maisons-Alfort, France) is a highly virulent strain isolated from a stork with severe neurological symptoms during the 1998 epidemic in Israel [62]. Working stocks of WNV IS-98-ST1 were generated by a single round of amplification in Vero E6 cells. Titers of virus stocks were determined by a standard serial dilution plaque assay in Vero E6 cells, and results expressed as PFU/mL [1,63].

### 2.2. Cloning of NS1

The NS1-coding gene from the WNV strain IS-98-ST1 was cloned in fusion with the coding sequence of a flexible linker (Gly-Ser-Gly) and an oligo-histidine tag (6× His) at the carboxy terminus. A similar tag has been previously added to the NS1^DENV^ protein, without any detrimental effect on its structural and biological properties [10,64]. The coding sequence of the C-terminal region of the E gene (72 nucleotides) was conserved at the 5’ end of the construct, as it contains the ER-targeting sequence and the host protease cleavage site. *Bam* HI and *Xho* I restriction sites were added at the 5’ and 3’ ends, respectively, using polymerase chain reaction (PCR) with the following primers: forward 5’-GGATCCAATACGGAATTCATGCAGCTGTTGAATTTTGACCTTCTCAAGCTTGCG-3’, and reverse 5’-CTCGAGTCACTAGTGGTGATGGTGATGATGGCCGCTTCCAGCATTCACTTGTGACTACG-3’. The fragment thus obtained was digested by *Bam* HI and *Xho* I (New England BioLabs, NEB™, Ipswich, MA, USA) and inserted into the *Bam* HI/*Xho* I-linearized pcDNA3.1 expression vector, using T4 DNA Ligase (NEB™), according to the manufacturer’s instructions. The chemically competent *E. coli* bacterial strain Top10 was used for plasmid construct propagation. Standard procedures were followed, except for the temperature of transformed bacterial cells propagation (28 °C). The quality of the plasmid construct, abbreviated as pNS1^WNV-IS98^ in the present study (Supplementary Figure S1), was verified by DNA sequencing. The empty pcDNA3.1 plasmid vector was used as negative control.

### 2.3. NS1 Production and Purification

One day before transfection, 4 × 10^6^ HEK-293T cells were seeded in 100 mm diameter Petri dishes in serum-reduced culture medium (DMEM, 1% FBS). On the following day, cells were transfected with 10 μg plasmid pNS1^WNV-IS98^, using Lipofectamine^®^ 2000 Transfection Reagent (Invitrogen™, Thermo Fisher Scientific, Carlsbad, CA, USA), following the manufacturer’s recommendations. Another batch of cells was transfected with empty pcDNA3.1 vector as negative control. At 6 h post-transfection, the medium was replaced by fresh DMEM supplemented with 1% FBS, and cells were further incubated at 37 °C, 5% CO_2_ for 3 days. The culture medium was then collected, filtered through 0.2 μm pores, and stored at 4 °C before NS1 purification. Fresh culture medium was added to the cells for a further 2-day period, then culture medium was again collected, filtered, and pooled with the previous batch stored at 4 °C.

His-tagged native protein sNS1 was purified by affinity chromatography on a nickel resin column. The pooled sNS1-containing culture medium was mixed with an equal volume of equilibration buffer (300 mM NaCl, 10 mM Imidazole, pH 7.4 in PBS). The sNS1-containing mix was then loaded onto a column of Ni-NTA resin (HisPur Ni-NTA resin, Thermo Fisher Scientific™) equilibrated with equilibration buffer. Resin was rinsed with wash buffer (PBS, 25 mM Imidazole, pH 7.4). Elution of the sNS1 protein was carried out with PBS containing 250 mM imidazole, pH 7.4. Eluate was dialyzed overnight against PBS at 4 °C, using SnakeSkin dialysis tubing (7 kDa MWCO, 22 mm; Thermo Fisher Scientific™). In parallel, the same putification protocol was applied to negative control samples (Ctrl^pcDNA3.1^), corresponding to the culture medium of empty pcDNA3.1-transfected HEK-293T cells. The sNS1 concentration was determined in the dialyzed samples, using Bradford protein microassay (protein assay dye reagent, Cat. #5000006, Bio-Rad™, Hercules, CA, USA). Absorbance was measured using the Victor^®^ microplate reader (Perkin Elmer, Waltham, MA, USA). All the samples were stored at −80 °C until further use.

### 2.4. Antibodies

Mouse monoclonal anti-5× His tag antibody was purchased from Qiagen (penta-His antibody, Cat. #34660). The mouse monoclonal anti-flavivirus NS1 antibody (Cat. #Ab214337) and the rabbit polyclonal anti-α-tubulin antibody (Cat. #Ab15246) were from Abcam. The fluorescent secondary antibodies Alexa Fluor^®^488-labeled goat anti-mouse IgG antibody (Cat. #A11029), Alexa Fluor^®^633-labeled goat anti-rabbit IgG antibody (Cat. #A21070), and Alexa Fluor^®^546-labeled goat anti-rabbit IgG antibody (Cat. #A11035) were from Invitrogen. The rabbit polyclonal antibody against domain III of E^WNV^ (anti-WNV-E^DIII^) was kindly provided by Dr. S. Lecollinet (ANSES-ENVA, Maisons-Alfort, France).

### 2.5. Protein Gel Electrophoresis and Western Blotting

Total cellular proteins were extracted using RIPA lysis buffer (100 μM NaCl, 20 mM Tris-HCl pH 7.4, 1 mM ethylenediaminetetraacetato (EDTA), 0.5% sodium deoxycholate, 1% sodium dodecyl sulfate (SDS)) containing a protease inhibitor cocktail (Halt™ protease inhibitor cocktail, Thermo Fisher Scientific™). Samples were denatured by heating at 95 °C for 5 min in an equal volume of two-fold concentrated loading buffer (2× loading buffer: 1 M Tris-HCl pH 6.8, containing 0.5 M Na_2_EDTA, 10% (*w*/*v*) SDS, 14.3 M β-mercaptoethanol, 20% (*v*/*v*) glycerol, and 0.04% (*w*/*v*) bromophenol blue). In parallel, heat treatment was omitted in corresponding samples, and proteins were denatured by mild treatment in SDS/β-mercaptoethanol loading buffer only, at room temperature (RT) for 10 min. Proteins were electrophoresed in SDS-containing 10% polyacrylamide gel (SDS-PAGE), and electrically transferred to nitrocellulose membranes (TransBlot^®^ system, Bio-Rad™). Blots were blocked for 1 h at RT in Tris-buffered saline (TBS) containing 0.05% Tween-20 (TBS-T) supplemented with 5% non-fat dry milk (blotto milk). Membranes were then incubated overnight at 4 °C with mouse monoclonal anti-flavivirus NS1 antibody (1:1000) or anti-5× His tag antibody (1:2000) in TBS-T supplemented with 1% blotto milk. After extensive washes with TBS-T, membranes were incubated with HRP-conjugated goat anti-mouse IgG (1:10,000 dilution, Sigma-Aldrich) for 1 h at RT. NS1 protein was detected using a chemiluminescent substrate (SuperSignal^®^ West Pico, Thermo Fisher Scientific™), and visualized using a ChemiDoc^®^ XRS+ system with Image Lab™ software (Bio-Rad™).

### 2.6. Confocal Fluorescence Microscopy

To study the expression and localization of NS1^WNV^ protein in plasmid-transfected or WNV-infected cells, or to monitor the NS1^WNV^ protein in sNS1^WNV^-treated cells, cells were fixed in PBS containing 4% paraformaldehyde for 30 min, then permeabilized with 0.5% Triton X-100 in PBS. Nonspecific signals were blocked with PBS containing 0.1% Triton X-100, 5% FBS (PBS–T-FBS). NS1 was detected using mouse monoclonal anti-flavivirus NS1 antibody (1:250 in PBS–T-FBS), followed by Alexa Fluor^®^488-labeled goat anti-mouse IgG antibody (1:500 in PBS–T-FBS). Expression and localization of viral E protein in infected cells (MOI 0.5) were monitored by reaction with rabbit polyclonal anti-WNV-E^DIII^ antibody (1:500 in PBS–T-FBS), followed by Alexa Fluor^®^633-labeled goat anti-rabbit IgG antibody (1:500 in PBS–T-FBS). In transfected cells, α-tubulin was detected with rabbit polyclonal anti-α-tubulin antibody (1:250 in PBS–T-FBS), followed by Alexa Fluor^®^633-labeled goat anti-rabbit IgG antibody (1:500 in PBS–T-FBS). In WNV-infected cells, labeling of α-tubulin was performed using Alexa Fluor^®^546-labeled goat anti-rabbit IgG antibody (1:500 in PBS–T-FBS). Actin microfilament (F-actin) staining was performed by reaction with rhodamine–phalloidin conjugate (R415, Invitrogen, Thermo Fisher Scientific™; 1:500 in PBS–T-FBS). Nuclei were stained with 4’,6-Diamidino-2-Phenylindole, dihydrochloride (DAPI) in PBS–T-FBS (1 μg/mL). Samples were examined under a Leica™ TCS SP5 laser scanning confocal microscope. All confocal images were acquired with strictly the same settings and analyzed using the LCS AF software. The *Z*-resolution was 400–600 nm, a value compatible with a nanofilament of 50–200 nm in diameter.

For kinetic analysis of actin network modifications occurring in sNS1^WNV^-treated cells, cell samples were collected at different times of incubation with sNS1^WNV^ at 37 °C (up to 24 h), fixed, and then stained with rhodamine–phalloidin. Negative controls consisted of mock-treated cells (i.e., cells incubated with samples from the culture medium of HEK-293T cells transfected with empty plasmid pcDNA3.1, subjected to the same chromatographic process as the culture supernatant of pNS1^WNV-IS98^-transfected cells). A total of 140 to 330 individual cells were examined by fluorescence microcopy in at least 5 to 10 separate microscopic fields. Images were acquired with the Leica™ TCS SP5 laser scanning confocal microscope, and cells with absent or disorganized actin microfilament networks were counted using the ImageJ program. The results were expressed as the percentage of total cells examined per field. Statistical analyses were performed using the Mann–Whitney test.

### 2.7. Flow Cytometry Analysis of Cell Attachment and Uptake of NS1^WNV^

Cells were seeded with complete media in 24- or 48-well plates to obtain a confluency of 70%–80% the following day. Cells (5 × 10^5^) were incubated with recombinant sNS1^WNV^ (10 μg/mL) or control sample (purified supernatant from pcDNA3.1-transfected HEK-293T cells) in PBS containing 10% FBS for 1 h on ice, and rinsed three times with ice-cold PBS. They were detached with PBS containing 0.01% trypsin and 1 mM Na_2_EDTA, then fixed with 4% paraformaldehyde in PBS for 30 min. To determine the level of cell surface-attached sNS1^WNV^, cells were incubated with mouse anti-NS1 antibody (1:250 in PBS) for 1 h on ice.

To analyze the cell internalization of sNS1^WNV^, cells were first incubated with sNS1^WNV^ for 1 h on ice to allow cell attachment, rinsed with ice-cold PBS, then transferred to 37 °C for 6 h to allow cellular internalization of sNS1^WNV^. Cells were detached with PBS containing 0.01% trypsin and 1 mM Na_2_EDTA, fixed in PBS containing 4% paraformaldehyde for 30 min, and permeabilized with PBS containing 0.1% saponin and 10% FBS for 15 min on ice. NS1 was reacted with mouse anti-NS1 antibody (1:250 in PBS) for 1 h on ice, as above. In both cases, the detection of sNS1^WNV^-positive cells was performed using a 1:500 dilution of AlexaFluor 488-conjugated goat anti-mouse IgG antibody for 1 h on ice. Cells were then rinsed 3 times with ice-cold PBS, resuspended in PBS containing 1% paraformaldehyde, and analyzed in a BD FACSCalibur™ flow cytometer.

### 2.8. Transmission Electron Microscopy

Samples of purified sNS1 were applied to a carbon film using the standard mica-carbon flotation technique, and negatively stained with 1% (*w*/*v*) uranyl acetate. They were examined under a JEOL (JEM-1200EXII) electron microscope at 100 kV. Images were acquired and analyzed by digital micrograph software (Gatan Inc., Pleasanton, CA, USA).

### 2.9. Transfections

On the day prior to transfection, 5 × 10^4^ Vero E6 cells or 8 × 10^4^ U-87MG and SH-SY5Y cells were seeded in 8-well plates (Nunc© Lab-Tek, Thermo Fisher Scientific, Carlsbad, CA, USA), and maintained in their respective media. On the next day, cells were transfected with 500 ng of pNS1^WNV-IS98^ plasmid or control empty pcDNA3.1, using Lipofectamine^®^ 2000 Transfection Reagent (Invitrogen™, Thermo Fisher Scientific), according to the manufacturer’s recommendations. At 6 h post-transfection, medium was replaced by fresh complete medium. Cells were fixed with 4% paraformaldehyde and processed for immunostaining.

### 2.10. Kinetics of WNV Replication and Virus Titration

WNV titration was performed as previously described [63]. In brief, VeroE6 cells were seeded at 1 × 10^5^ cells per well, and SH-SY5Y and U-87MG cells were seeded at 2 × 10^5^ cells per well of 48-well plates, in DMEM supplemented with 10% FBS (Gibco). The next day, the cells were incubated with sNS1^WNV^ (10 μg/mL) or with an aliquot of control sample from empty plasmid pcDNA3.1-transfected cells for 5 h at 37 °C with 5% CO_2_, before or concomitantly to virus addition. WNV-IS98 inoculum at different MOI (in DMEM supplemented with 2% FBS for Vero E6 and U-87MG, and in DMEM/F12 supplemented with 2% FBS for SH-SY5Y) was added to the wells and incubated for 2 h at 37 °C and 5% CO_2_. Cells were rinsed twice with fresh DMEM, then further incubated at 37 °C and 5% CO_2_ in DMEM supplemented with 10% FBS for Vero E6 and U-87MG, and in DMEM/F12 supplemented with 2% FBS for SH-SY5Y. Cell supernatants were collected at different times post-infection, and virus progeny titers (expressed as Log_10_TCID_50_/mL) were determined by end point dilution assays in Vero E6 cells, using the Reed and Muench method [1,63].

## 3. Results

### 3.1. Expression and Characterization of Intracellular and Secreted Form of NS1^WNV^ in Mammalian Cells

Recombinant His-tagged NS1 protein was produced in pNS1^WNV-IS98^-transfected HEK-293T cells and isolated from the cell culture medium as the secreted form of NS1 protein (abbreviated sNS1^WNV^). HEK-293T cell lysates and culture supernatants were analyzed by SDS-PAGE and Western blotting, using the standard procedure of protein denaturation by SDS treatment at 95 °C, or, alternatively, heat-denaturation was omitted to preserve the oligomeric structures. In the latter case, samples were only treated with SDS at room temperature, with no chemical cross-linker.

**Figure 1:**
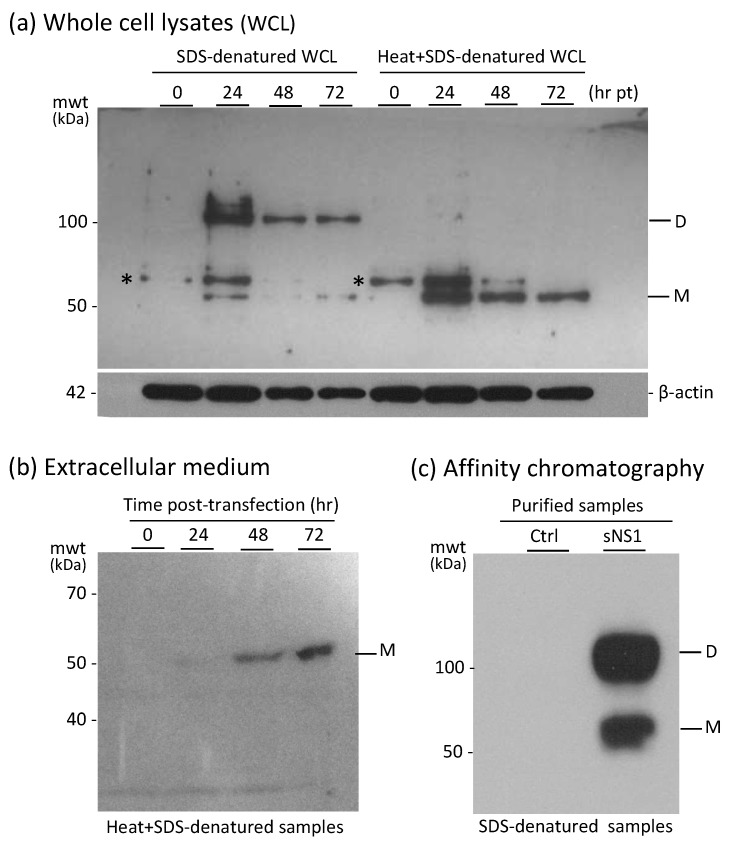
Expression and secretion of the recombinant NS1^WNV^ protein into the culture medium of pNS1^WNV-IS98^-transfected HEK-293T cells. (**a**) Kinetics of intracellular expression of NS1^WNV^. Whole cell lysates (WCL) were denatured in SDS/β-mercaptoethanol-containing loading buffer at room temperature (RT) to preserve the oligomeric status of proteins (leftmost lanes), or subjected to the usual heat denaturation at 95 °C for 5 min in loading buffer (rightmost lanes). Proteins were analyzed by SDS-PAGE and Western blotting. H6-tagged NS1^WNV^ protein was detected using anti-His6 tag antibody. Note: * = the protein band migrating with an apparent mwt of 58 kDa and visible in both control and NS1-expressing samples might correspond to the superimposition of an anti-His6-tag antibody-reacting cellular protein and a post-translationally modified form of the NS1^WNV^ protein. (**b**) Secretion of sNS1^WNV^ protein. Samples from the culture medium of pNS1^WNV-98^-transfected cells were collected at the indicated times post-transfection (pt), heat-denatured in SDS/β-mercaptoethanol-containing loading buffer, and analyzed by SDS-PAGE and Western blotting. (**c**) Purification and oligomeric status of sNS1^WNV^. Extracellular H6-tagged sNS1^WNV^, isolated by affinity chromatography on Ni_2_^+^-agarose column, was analyzed by SDS-PAGE and Western blotting after mild denaturation in SDS/β-mercaptoethanol-containing loading buffer at RT. Abbreviations: D = sNS1^WNV^ dimers; M = sNS1^WNV^ monomers; Ctrl = negative control samples; mwt = molecular weight (kDa).

The majority of the intracellular NS1^WNV^ protein occurred as dimers of 110 kDa at 24 h post-transfection (pt; Figure 1a). The secreted sNS1^WNV^ protein (55 kDa) was detected in low amounts in the culture supernatant as early as 24 h pt, and the maximum extracellular release was observed at 72 h pt (Figure 1b). No apparent cytotoxicity was observed for the sNS1^WNV^-producer cells up to 72 h pt, as shown by MTT assays. After affinity purification, the majority of sNS1^WNV^ protein in non-heated, SDS-treated samples migrated as dimers, but monomers were also present in significant amounts (Figure 1c).

After purification by affinity chromatography, the final recovery of sNS1^WNV^ was about 2 μg protein per 10^6^ cells over a 5 day culture period (2.15 ± 0.45 pg/cell; mean ± SD, *n* = 4). In the absence of an available antibody specific to sNS1^WNV^ hexamers, their proportion was impossible to assess.

**Figure 2:**
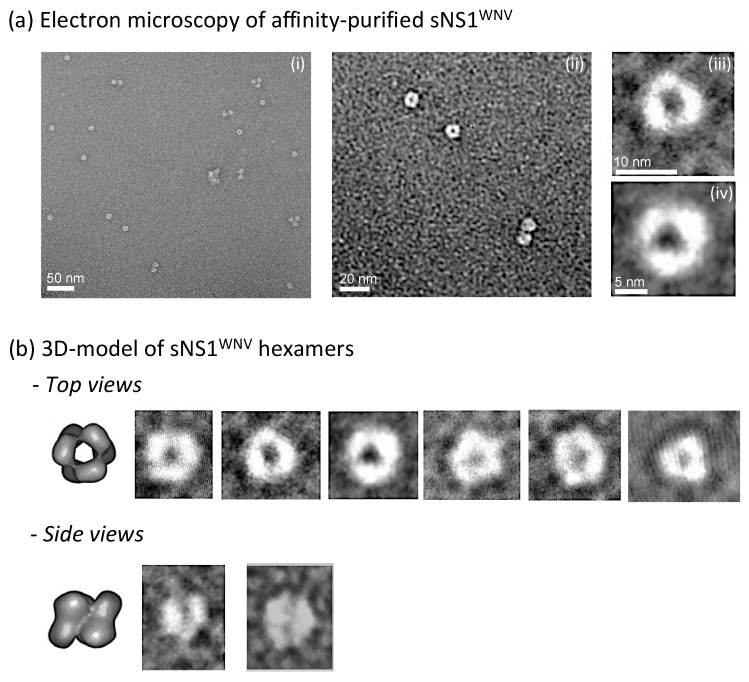
Electron microscopy control of extracellular sNS1^WNV^. Samples from the extracellular medium of pNS1^WNV-IS98^-transfected HEK-293T cells were purified by affinity chromatography on a Ni_2_^+^-agarose column, and the oligomeric status of sNS1^WNV^ proteins was verified by electron microscopy (EM). (**a**) EM images of negatively stained samples observed at low (**i**) and high (**ii**) magnifications. (**iii**,**iv**) Enlargements of individual sNS1^WNV^ hexamers. (**b**) Three-dimensional model previously proposed for NS1^DENV^ [4], and comparison with enlargements of sNS1^WNV^ hexamers presented from different incidences—top and side views.

Electron microscopic (EM) observation of negatively stained sNS1 samples purified by affinity chromatography showed that the secreted recombinant sNS1^WNV^ protein presented the same structural characteristics as those previously reported for other flaviviral NS1 proteins [10,65]. The top view and side view EM images of our sNS1^WNV^ protein (Figure 2) were consistent with a hexameric structure, as previously shown for the sNS1 protein of dengue virus (NS1^DENV^) using 3D reconstruction [4]. The fact that dimers—but no hexamers—were detected by SDS-PAGE analysis of non-heated, SDS-treated samples of sNS1^WNV^ (Figure 1c), even in gels of low acrylamide concentration, implied that sNS1^WNV^ dimers interacted via noncovalent links more resistant to SDS dissociation, compared to the other links involved in the whole hexameric structure.

This was in agreement with a previous study showing that the hexamers of NS1^DENV^ are unstable in non-ionic detergents, whereas the interfaces between the dimeric subunits are very narrow and contribute to the stabilization of the dimers [4]. Our result confirmed that NS1 dimers constitute the building blocks of the NS1 hexameric scaffold, and that three dimers are held together by weak hydrophobic interaction to form one hexamer [4].

### 3.2. Cell Attachment and Uptake of sNS1^WNV^

**Figure 3:**
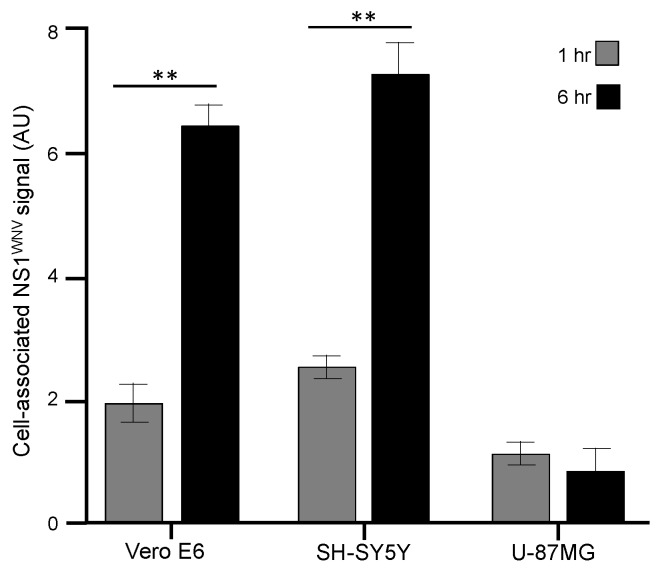
Cell attachment and uptake of recombinant sNS1^WNV^ by different cell types. Aliquots of extracellular, H_6_-tagged sNS1^WNV^ protein, released by pNS1^WNV-IS98^-transfected HEK-293T cells and purified by affinity chromatography, were added to the cell culture media of Vero E6, SH-SY5Y, and U-87MG cells, at a final concentration of 10 μg/mL. In one series of samples, cells were collected after 1 h incubation on ice (gray bars) and cell-bound NS1^WNV^ was detected and quantified by flow cytometry. In another series (black bars), cells were first incubated on ice for 1 h, then transferred to 37 °C for 6 h, permeabilized, and intracellular NS1^WNV^ was evaluated by flow cytometry. Control mock-treated cells were incubated with samples from the culture medium of HEK-293T cells transfected with empty plasmid pcDNA3.1, subjected to the same chromatographic process as the culture supernatant of pNS1^WNV-IS98^-transfected cells. The mean fluorescence intensity (MFI) values shown in the bar graph (expressed in arbitrary units; AU), represented the mean values (*m ± SD*) of duplicate samples from two separate experiments, corrected by subtraction of the values of the signal obtained with mock-treated cells. Statistical analyses were performed using the Mann–Whitney test. Note: ** = *P* ≤ 0.01.

It has been reported that sNS1^DENV^ can bind to the cell membrane of epithelial and fibroblast cells [15], and that NS1^WNV^ binds to HEK-293T cells, followed by its internalization within 6 h [16]. Therefore, we compared the cell attachment and cellular uptake of sNS1^WNV^ in three different cell types: (i) Vero E6, a simian kidney epithelial cell line; (ii) SH-SY5Y, a human neuroblastoma cell line; and (iii) U-87MG, a human astrocytoma cell line. All three cell lines are permissive to WNV infection. The cells were incubated with sNS1^WNV^ at 10 μg/mL for 1 h and 6 h, respectively, and analyzed by flow cytometry for cell-bound sNS1^WNV^ (1 h samples, maintained at low temperature) and intracellular NS1^WNV^ (6 h samples, subjected to temperature increase and cell permeabilization). After a 1 h incubation period, sNS1^WNV^ proteins were detectable on the surface of non-permeabilized Vero E6 and SH-SY5Y cells, and in significantly lower amounts on the surface of U-87MG cells (Figure 3). After 6 h incubation, sNS1^WNV^ was efficiently internalized into Vero E6 and SH-SY5Y cells, but not in U87MG cells (Figure 3). These results implied that even though sNS1^WNV^ could bind to a variety of cells, as previously reported [10,14,15,18], the cellular uptake of sNS1^WNV^ was cell-type-dependent.

### 3.3. Cytoskeletal Modifications Associated with sNS1^WNV^ Internalization

**Figure 4:**
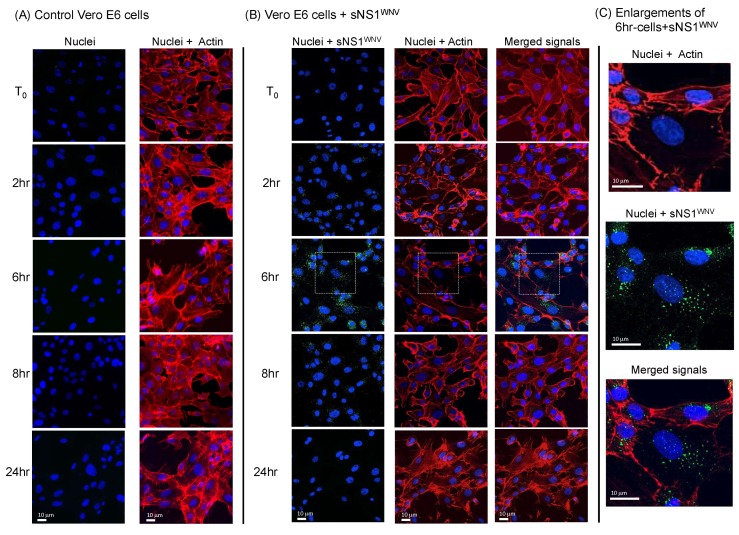
Time-course analysis of the cellular uptake of sNS1^WNV^ by Vero E6 cells. Extracellular, soluble H_6_-tagged sNS1^WNV^ protein, released by pNS1^WNV-IS98^-transfected HEK-293T cells and purified by affinity chromatography, was added to the cell culture medium of Vero E6 cells at 10 μg/mL. Control, mock-treated cells consisted of cells incubated with aliquots from the culture medium of HEK-293T cells transfected with empty plasmid pcDNA3.1, and were subjected to the same chromatographic process as the culture supernatant of pNS1^WNV-IS98^-transfected cells. Cells were fixed at different periods of incubation at 37 °C in the presence of sNS1^WNV^ (0 to 24 h, as indicated on the left side of panels (**A**,**B**), and analyzed by confocal fluorescence microscopy. (**A**) Control Vero E6 cells. (**B**) Vero E6 cells incubated with sNS1^WNV^. (**C**) Enlargements of microscopic images of sNS1^WNV^-treated cells (6 h incubation time, delineated by a white square). Actin microfilaments (red) were visualized using rhodamine–phalloidin, nuclei (blue) were visualized using dihydrochloride (DAPI) reagent, and sNS1^WNV^ protein (green) was immunolabeled using anti-NS1^WNV^ rabbit antibody. Note that images in panels (**A**–**C**) show the same areas at a given time point.

We then analyzed the effect of the internalization of sNS1^WNV^ on the cytoskeleton of Vero E6, SH-SY5Y, and U-87MG cells. Recombinant sNS1^WNV^ was added to the culture medium at 10 μg/mL, and the cells were harvested and fixed after 2 h, 6 h, 8 h, and 24 h. The presence of intracellular NS1^WNV^ and the morphological status of the actin and tubulin networks were analyzed by confocal fluorescence microscopy. The NS1^WNV^ signal was detected in sNS1^WNV^-treated Vero E6 cells as early as 2 h post-incubation, reaching a maximum at 6 h (Figure 4A). NS1^WNV^ was still visible at 8 h, but no longer detectable at 24 h (Figure 4A).

**Figure 5:**
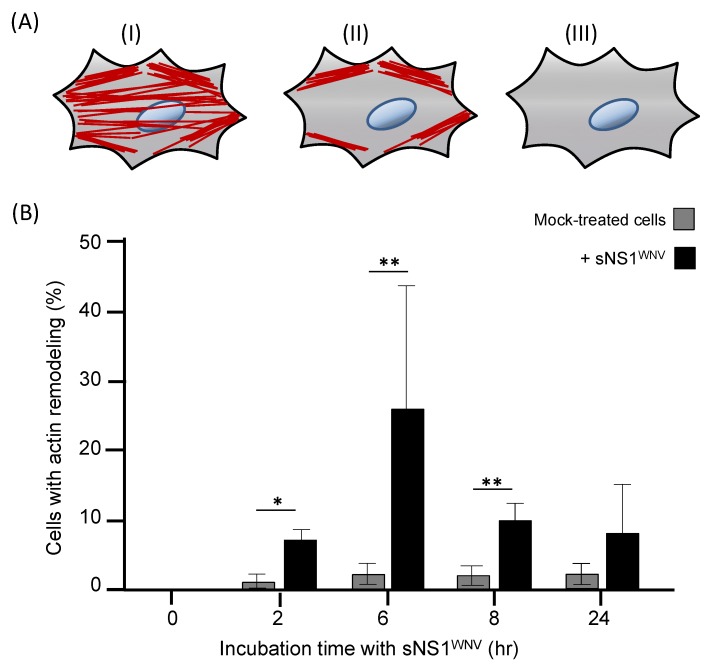
Kinetics of actin remodeling in sNS1^WNV^-treated Vero E6 cells. Extracellular, soluble H_6_-tagged sNS1^WNV^ protein, released by pNS1^WNV-IS98^-transfected HEK-293T cells and purified by affinity chromatography, was added to the cell culture medium of Vero E6 cells, at a final concentration of 10 μg/mL. Control, mock-treated cells consisted of cells incubated with samples from the culture medium of HEK-293T cells transfected with empty plasmid pcDNA3.1, subjected to the same chromatographic process as the culture supernatant of pNS1^WNV-IS98^-transfected cells. At different times of incubation with sNS1^WNV^ at 37 °C (0 to 24 h), cells were fixed and stained with rhodamine–phalloidin. (**A**) Schematic representation of cells grouped according to different F-actin morphologies. (**I**) Cells with organized actin fibers, as observed in the majority of mock-treated cells. (**II**) Cells showing reduced number of organized actin filaments, and notably the absence of perinuclear actin network. (**III**) Cells showing total absence of actin filaments. (**B**) Cell counts. Cells with alterations of the actin network as defined above were counted using the ImageJ program, and the results of groups (II) and (III) were pooled and expressed as the percentage of the total number of cells examined per field. A total of 140–330 individual cells were examined by fluorescence microcopy in at least 5–10 separate microscopic fields. Data presented in the bar graph are mean values (*m ± SD*) obtained from three separate experiments. Statistical analyses were performed using the Mann–Whitney test, with *p* ≤ 0.05 (*); *p* ≤ 0.01 (**).

In parallel, a significant remodeling of the cellular actin network was observed between 6 h and 8 h (Figure 4B). These alterations were transient and followed the evolution of the NS1^WNV^ content; after a maximum disorganization at 6–8 h, the actin network was completely restored at 24 h (Figure 4B and Figure 5). This suggested that the extent of actin depolymerization correlated with the level of intracellular NS1^WNV^. A direct negative effect of NS1^WNV^ on actin biosynthesis was unlikely, as shown by the absence of significant change in actin levels over time in NS1^WNV^-expressing HEK-293T cells (refer to Figure 1a).

Our observations, therefore, implied that the viral protein sNS1^WNV^ alone had a transient negative effect on the actin network of epithelial cells. The effect on actin was apparently specific to epithelial cells, as no remodeling of the actin filament network was visible in sNS1^WNV^-treated neuronal cell SH-SY5Y or glial cell U-87MG (not shown). No detectable alteration of the tubulin network was observed in any of the sNS1^WNV^-treated cell types, namely, Vero E6, SH-SY5Y, or U-87MG (Supplementary Figure S2).

### 3.4. Presence of NS1^WNV^ Protein in Actin-Containing, TNT-Like Nanofilaments Protruding from NS1^WNV^-Expressing and WNV-Infected Cells

**Figure 6:**
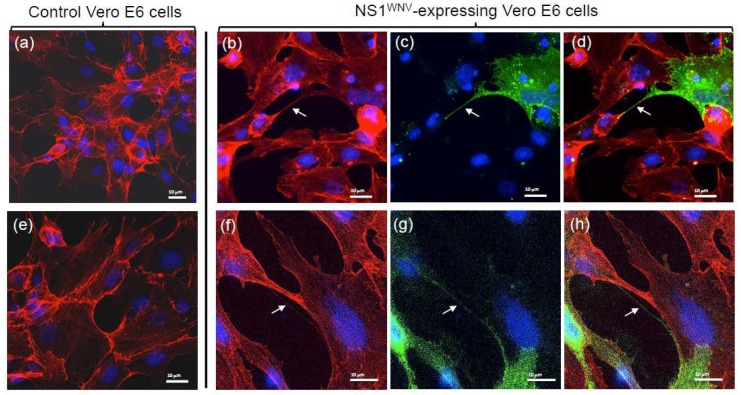
Actin network in recombinant NS1^WNV^-expressing Vero E6 cells. Vero E6 cells were transfected with control empty plasmid pcDNA3.1 (**a**,**e**), or with pNS1^WNV-IS98^ (**b**–**d**,**f**–**h**). Cells were analyzed at 24 h pt by confocal fluorescence microscopy, using rhodamine–phalloidin for the visualization of actin filaments and specific rabbit antibody for the immunolabeling of NS1^WNV^. (**a**,**b**,**e**,**f**) Actin signal; (**c**,**g**) NS1^WNV^ signal; (**d**,**h**) merged signals. Nuclei were stained in blue using DAPI reagent. (**a**–**d**) Low magnification; (**e**–**h**) high magnification. Arrows point to actin- and NS1-containing cellular TNT-like projections.

Since the cell attachment and cellular uptake of sNS1^WNV^ differed for the three cell types, the possible effects of endogenous NS1^WNV^ on the cytoskeletal network of Vero E6, SH-SY5Y, and U-87MG cells were analyzed after transfection with pNS1^WNV-IS98^ plasmid. The actin network disorganization was less obvious in NS1^WNV^-expressing Vero E6 cells compared to cells treated with exogenous sNS1^WNV^ (Figure 6; compare to Figure 4). Only Vero E6 cells overexpressing NS1^WNV^ protein showed less distinct stress fibers compared to mock-transfected cells (Figure 6a–d).

**Figure 7:**
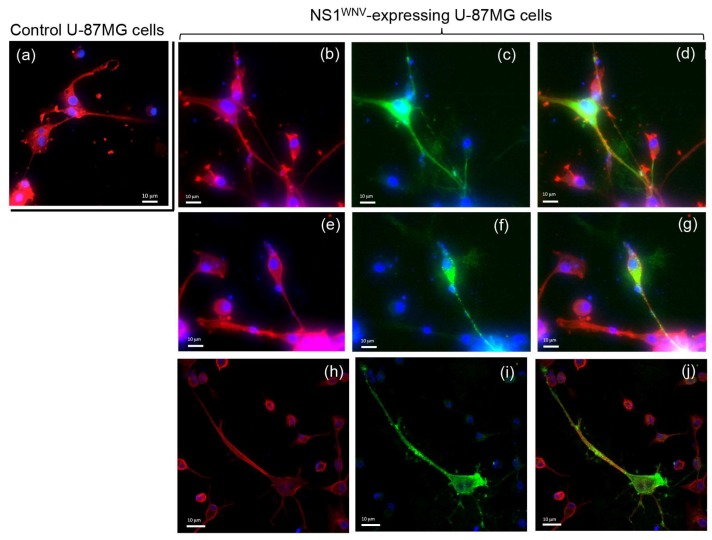
Actin network in recombinant NS1^WNV^-expressing human glial cells U-87MG. U-MG87 cells were transfected with control empty plasmid pcDNA3.1 (**a**) or with pNS1^WNV-IS98^ (**b**–**j**) and analyzed at 24 h pt by confocal fluorescence microscopy, using rhodamine–phalloidin for the visualization of actin filaments, and specific rabbit antibody for the immunolabeling of NS1^WNV^. (**b**,**e**,**h**) Actin signal; (**c**,**f**,**i**) NS1^WNV^ signal; (**d**,**g**,**j**) merged signals. Nuclei were stained in blue using DAPI reagent. The panels presented in this figure show microscopic fields observed at different magnifications. Note the coexistence of actin and NS1^WNV^ signals in the cellular projections.

Interestingly, NS1^WNV^-expressing Vero E6 cells produced thin and long projections joining one cell to another and containing both actin and NS1^WNV^ proteins (Figure 6b,f–h). These filamentous projections resembled the thin, actin-rich membrane channels referred to as tunneling nanotubes (TNTs), involved in intercellular communication and exchange [40,41,43,44]. In U-87MG cells, TNT-like filaments were observed in control, mock-transfected cells (Figure 7a), but they seemed to occur with a higher frequency in NS1^WNV^-expressing cells (Figure 7b). NS1^WNV^-expressing SH-SY5Y cells also showed filamentous protrusions containing actin and NS1^WNV^, but these structures appeared to be thicker and shorter compared to the TNT-like nanofilaments observed in Vero E6 or U-87MG cells (Supplementary Figure S3).

**Figure 8:**
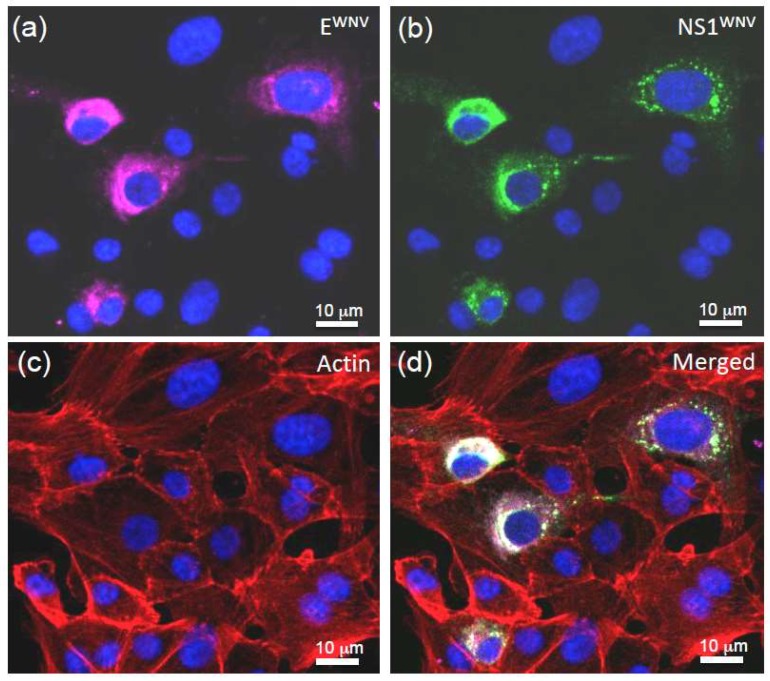
TNT-like structures in WNV-infected cells. Vero E6 cells were infected with WNV at MOI 0.5, and harvested at 18 h post-infection. After fixation and permeabilization, the cells were reacted with rhodamine–phalloidin to visualize the actin network, with an antibody against NS1^WNV^, and with anti-glycoprotein-E^WNV^ antibody to localize the virus and identify the WNV-infected cells. (**a**) E^WNV^ envelope glycoprotein (purple); (**b**) NS1^WNV^ protein (green); (**c**) actin (red); (**d**) merged signals. Nuclei were stained in blue using DAPI reagent. Note that the thin filament connecting a WNV-infected cell to a non-infected cell contained both NS1^WNV^ and E^WNV^.

We next determined whether the TNT-like nanofilaments also occurred during WNV infection. Vero E6 cells were infected with WNV at relatively low multiplicity (MOI 0.5), and the cells were fixed at 18 h post-infection for analysis by confocal fluorescence microscopy. Actin-containing filamentous projections emanating from WNV-infected cells were visible (Figure 8), and, interestingly, they contained both the viral envelope glycoprotein E^WNV^ (Figure 8a) and the non-structural protein NS1^WNV^ (Figure 8b). In some cases, we observed that TNT-like structures made connections to both WNV-infected and non-infected cells, and certain images suggested a possible transfer of NS1^WNV^ to non-infected cells (Figure 8c,d). These observations were consistent with the results of an earlier study on WNV-infected Vero cells examined at the late stages of infection, showing the proliferation of filopodia associated with actin filament thickening, and the presence of mature WNV virions in some of these filopodia [66].

### 3.5. NS1^WNV^ and Virus Infectiveness

Considering that NS1^WNV^ was secreted in the extracellular milieu of WNV-infected cells and was also susceptible to spread from cell to cell via actin-containing cellular nanofilaments, we analyzed the possible impact of NS1^WNV^ on the WNV life cycle. Vero E6, SH-SY5Y, and U-87MG cells were incubated with sNS1^WNV^ added to the culture medium 5 h prior to, or concomitantly with, the virus inoculum, used at MOI 0.01, 0.1, and 1, respectively. WNV-infected cells were then monitored for late cytopathic effects, and virus replication and progeny yields were determined by end point dilution assays in Vero E6 cells [1,63]. Interestingly, sNS1^WNV^ did not significantly affect any of these parameters in all three cell types (Supplementary Figures S4 and S5). This result was somehow expected for U-87MG cells, considering that these cells did not internalize sNS1^WNV^ in detectable amounts, but not for Vero E6 and SH-SY5Y cells, which showed an efficient sNS1^WNV^ uptake (refer to Figure 3). Although these negative data were obtained from in vitro experiments, they might have important implications regarding the role of NS1 in WNV pathogenicity.

## 4. Discussion

The NS1 protein used in this study was derived from WNV-IS-98-ST1, a neurovirulent strain of lineage 1 that has been well-characterized and is highly pathogenic in animal models [62,67]. Two forms of recombinant NS1^WNV^ protein were tested on cells: the exogenous, secreted form (sNS1^WNV^) added to the cell culture medium; and endogenous NS1^WNV^, the recombinant protein expressed in pNS1^WNV-IS98^-transfected cells. The possible cytological effects of NS1^WNV^ were analyzed using three different types of cells: Vero E6, SH-SY5Y, and U-87MG. The rationale for this choice was the following: (i) simian kidney epithelial cells Vero E6 are the most widely used host cell line for WNV propagation and titration; (ii) similar to Vero E6 cells, SH-SY5Y cells are permissive to WNV infection [68]; (iii) human neuroblastoma cells SH-SY5Y and human astrocytoma cells U-87MG represented valuable cellular models to study the sNS1^WNV^-mediated neurovirulence.

Cell attachment and uptake have been well-documented for sNS1^DENV^ compared to the other flaviviral NS1 proteins. It is generally accepted that sNS1^DENV^ binds to the cell plasma membrane via interactions with heparan sulfate or chondroitin sulfate E, and that TLRs can serve as alternative receptors [10,15,16,17,18]. After cell attachment, sNS1^DENV^ follows the endocytic pathway and accumulates in the late endosomal compartment [14]. For the NS1^WNV^ protein, we observed that the exogenous sNS1^WNV^ was efficiently internalized into Vero E6 and SH-SY5Y cells, reaching a maximum at 6 h incubation at 37 °C; it was not efficiently internalized into U-87MG. The cellular uptake of sNS1^WNV^, therefore, varied with the cell type.

The addition of sNS1^WNV^ to the cell culture medium was associated with a significant remodeling of the actin filament network, and the pattern suggested the depolymerization of the F-actin. This phenomenon was transient as the actin network progressively reformed in parallel with the decrease of the intracellular NS1^WNV^ signal. The alteration of the actin network was only observed in Vero E6 cells and not in the other two cell lines, implying that there was no direct correlation between the cell internalization of sNS1^WNV^ and alteration of the actin network. The absence of actin effect in sNS1^WNV^-treated SH-SY5Y cells, although sNS1^WNV^ was internalized with the same efficiency as in Vero E6 cells (refer to Figure 3), suggested that the sNS1^WNV^-associated actin remodeling was cell-type-dependent.

The cell type specificity of actin remodeling could be related to variable expression of surface molecules on the different cell types. Thus, tissue-specific variations in the cell binding of flaviviral NS1 proteins, as well as in the profile of response to inflammatory cytokines in the context of flaviviral infection, have been reported [69,70]. In terms of intracellular mechanism, a significant activation of RhoA by phosphorylation has been found in both DENV-infected and NS1^DENV^-treated human dermal microvascular endothelial cells, suggesting that the increased endothelial permeability observed during DENV infection or NS1^DENV^ treatment would be due, at least in part, to RhoA phosphorylation [71]. A quantitative proteomic analysis of brain tissue in WNV-infected mice has shown that several proteins related to the actin cytoskeleton and Rho GTPase signaling pathway were significantly modified during the course of neuroinvasive WNV infection [72]. The role of molecules of the Rho GTPase signaling pathway in cytoskeleton rearrangement, and the consequences on entry, replication, and release of many viruses, including WNV, have been well-documented [28,29]. It is, therefore, plausible (i) that the binding of sNS1^WNV^ to a G-protein-coupled receptor present on the surface of certain cells could activate the cascade of the Rho GTPase signaling pathway; or alternatively (ii) that the intracellular localization of cell-internalized sNS1^WNV^ would vary with the cell type, and, in some particular cases, would allow a direct interaction of sNS1^WNV^ with molecules of the Rho GTPase pathway. In both scenarios, the regulation of actin polymerization would be significantly affected.

The influence of NS1^WNV^ on the cytoskeletal network was then analyzed using endogenous NS1^WNV^. The pattern shown by NS1^WNV^-expressing Vero E6 cells significantly differed from that observed in cells treated with exogenous sNS1^WNV^, and only discrete changes in the actin network were observed (compare Figure 4 and Figure 6). This result supported the first hypothesis formulated above, and suggested that the NS1^WNV^-mediated modulation of the actin network in epithelial cells would require the attachment of sNS1^WNV^ to the cell surface and its transit via the endocytic pathway.

Examination of NS1^WNV^-expressing Vero E6 and U-87MG cells showed long and thin projections, forming intercellular bridges harboring both actin and NS1^WNV^ proteins. These nanofilaments presented morphological similarities with TNTs, the actin-rich tunneling nanotubes that form intercellular channels [40,41,43,44]. In contrast, NS1^WNV^-expressing SH-SY5Y cells showed thicker and shorter protrusions, but these filamentous structures also contained actin and NS1^WNV^. The structural characteristics of the SH-SY5Y protrusions were consistent with the recently elucidated structure of neuronal TNTs, which are composed of bundles of individual TNTs held together by N-cadherin fibers [45]. TNTs have been observed in higher numbers in WNV-infected Vero E6 cells compared to mock-infected cells [66]. Although our study lacked quantitative data, the TNT-like nanofilaments seemed to occur with a higher frequency in NS1^WNV^-expressing cells compared to control cells. Specifically designed methodologies would be necessary to quantify these NS1^WNV^-containing, TNT-like structures and determine a possible correlation between their density per cell and the level of NS1^WNV^ expression.

The TNTs that reached out to connect to neighboring cells, as observed in NS1^WNV^-expressing and WNV-infected cells, represented a well-designed launching site for the cell-to-cell transfer of NS1^WNV^. It was, therefore, tempting to hypothesize that the extracellular form of NS1^WNV^ (capable of cell entry), the intra-TNT form of NS1^WNV^ (potentially apt to cell-to-cell trafficking), or both, could prime uninfected cells present in this region, and increase their permissiveness to newly released WNV virions, as described for HIV-1 and Nef-induced filopodia [73,74]. We tested this hypothesis and found that exogenous sNS1^WNV^ (added to the culture medium of WNV-infected cells prior to, or simultaneously with, the virus) did not significantly change the late cytopathic effects, the replication kinetics, or the virus progeny yields in any of the three cell types. The absence of a direct effect of exogenous sNS1^WNV^ on the WNV infectiveness in vitro seemed to exclude its role as a virus facilitating factor or enhancer. However, the eventuality of such a role is still conceivable for endogenous NS1^WNV^ transiting through TNTs.

Another hypothesis might explain the possible advantage for the virus of the frequent occurrence of NS1^WNV^-associated TNT-like nanofilaments, and even their proliferation, as previously observed [66]: TNTs could facilitate the passage of WNV virions from one cell to another, so as to avoid their exposure to the extracellular environment, a mechanism of cell-to-cell transmission that would be similar to that of many other viruses [46,47,48,49,50,51,52,53,54,55,56,57,58]. Our observations that NS1^WNV^ and the envelope glycoprotein E^WNV^ coexisted in the filamentous bridges between WNV-infected and non-infected cells supported this hypothesis. Alternatively, TNTs could be used for the extracellular release of the WNV progeny, a mechanism which has previously been envisaged. Analysis of WNV-infected Vero cells analyses by transmission EM and atomic force microscopy suggested the exit of virus particles via the filopodia [66].

Regardless of the exact functions of the actin- and NS1^WNV^-containing TNT-like nanofilaments observed in NS1^WNV^-expressing and WNV-infected cells, our data clearly showed that recombinant NS1^WNV^ protein alone, without any other WNV protein, was capable of modulating—either directly or indirectly via host-cell intermediate factor(s)—the actin network of epithelial cells. These results should be considered in future studies performed in the context of natural infections with WNV, as they might provide further clues to elucidate the molecular mechanisms of the NS1-mediated disruption of endothelial cell monolayers, and the subsequent vascular leakage, as observed with sNS1^DENV^ [17,24,75].

## Supplementary Materials

Supplementary materials can be found at https://www.mdpi.com/1999-4915/11/10/901/s1.
