# Supplementary material for: Remodeling of the Actin Network Associated with the Non-Structural Protein 1 (NS1) of West Nile Virus and Formation of NS1-Containing Tunneling Nanotubes
Source: Viruses. 2019 Sep 27;11(10):901. doi: 10.3390/v11100901 (PMC6832617; doi:10.3390/v11100901)
Supplement: Supplementary file 1 [file viruses-11-00901-s001.pdf]

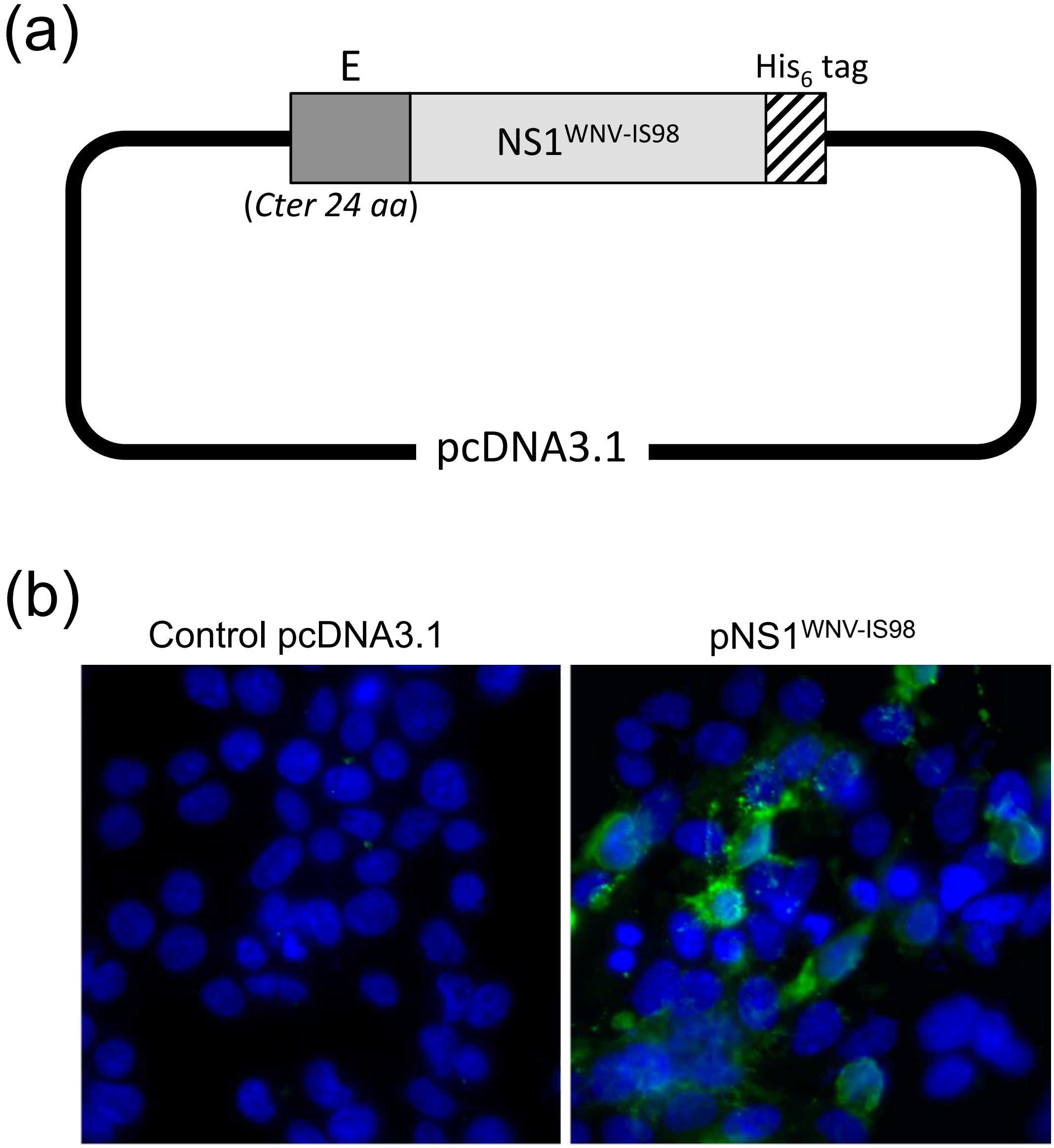

Supplementary Fig. S1. ***(a), Scheme of the plasmid construct used for the expression of NS1^{WNV} in HEK-293T cells***. The gene coding for the NS1 protein of WNV (strain IS-98-ST1) was cloned between the *Bam* HI and *Xho* I sites of the pcDNA3.1 expression plasmid. A nucleotide sequence coding for a flexible linker (oligopeptide G-S-G) was inserted at the C-terminus of NS1^{WNV}, upstream to the sequence coding for the oligo-histidine tag (H_6). The last 72 nucleotides of the E^{WNV} gene, coding for the 24 amino acids of the viral polyprotein upstream to the NS1^{WNV} gene coding sequence, were kept in this construct, as they carry the addressing signal to the endothelial reticulum compartment and the protease cleavage site. ***(b), Expression of the recombinant protein NS1^{WNV} in pNS1$^{WNV-IS98}$-transfected HEK-293T cells***. Intracellular NS1^{WNV} was detected by immunofluorescence confocal microscopy at 24hr post-transfection, using anti-H_6 tag antibody (*right panel*). Control cells consisted of HEK-293T transfected with empty plasmid pcDNA3.1 (*left panel*). Nuclei were stained in blue with DAPI.

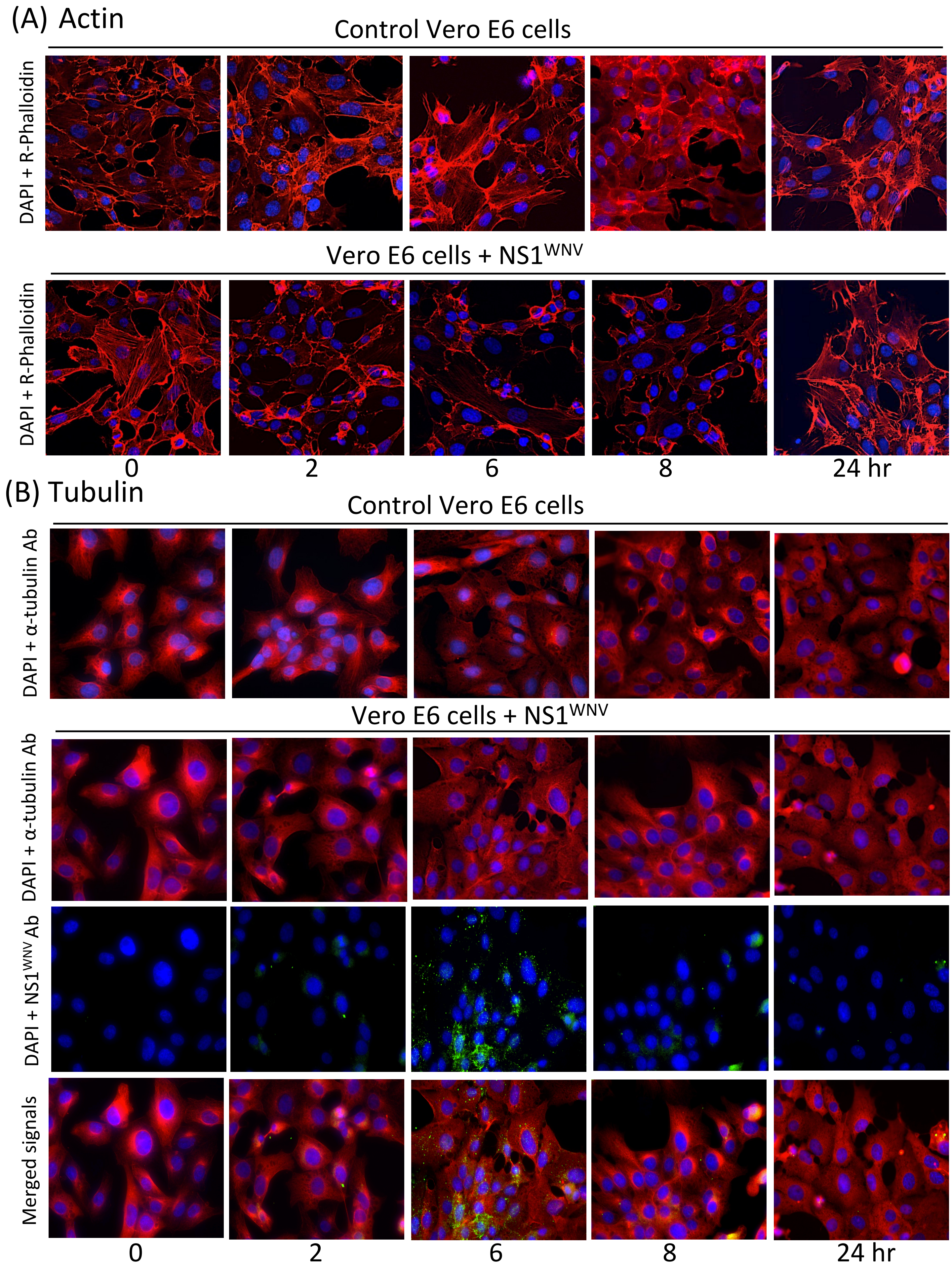
(A) Actin
Control Vero E6 cells
DAPI + R-Phalloidin
Vero E6 cells + NS1WNV
DAPI + R-Phalloidin
0
2
6
8
24 hr
(B) Tubulin
Control Vero E6 cells
DAPI + α-tubulin Ab
Vero E6 cells + NS1WNV
DAPI + α-tubulin Ab
DAPI + NS1WNV Ab
Merged signals
0
2
6
8
24 hr

Supplementary Fig. S2. ***Comparative evolution of cellular actin (A) and tubulin (B) networks in sNS1^{WNV}-treated cells***. Extracellular, soluble H6-tagged sNS1^{WNV} protein, released by pNS1WNV-IS98-transfected HEK-293T cells and purified by affinity chromatography, was added to the cell culture medium of Vero E6 cells at 10µg/mL. Control, mock-treated cells consisted of cells incubated with aliquots from the culture medium of HEK-293T cells transfected with empty plasmid pcDNA3.1, and subjected to the same chromatographic process as the culture supernatant of pNS1$^{WNV\text{-}IS98}$-transfected cells. Cells were fixed at different periods of incubation at 37°C in the presence of sNS1^{WNV} (0 to 24hr, as indicated at the bottom of panels A & B), and analyzed by confocal fluorescence microscopy. **(A)**, Actin filaments in control and sNS1^{WNV}-treated cells were labeled in fluorescent red with rhodamine-phalloidin. **(B)**, Control and sNS1^{WNV}-treated cells were reacted with rhodamine-phalloidin for the visualization of actin filaments, and with anti-α-tubulin antibody (Ab) and anti-NS1^{WNV} antibody for the immunostaining of α-tubulin and NS1^{WNV} protein, respectively. Nuclei were stained in blue using DAPI reagent. The merged signals are shown in the bottom row. All images were acquired with the same settings and at the same magnification.

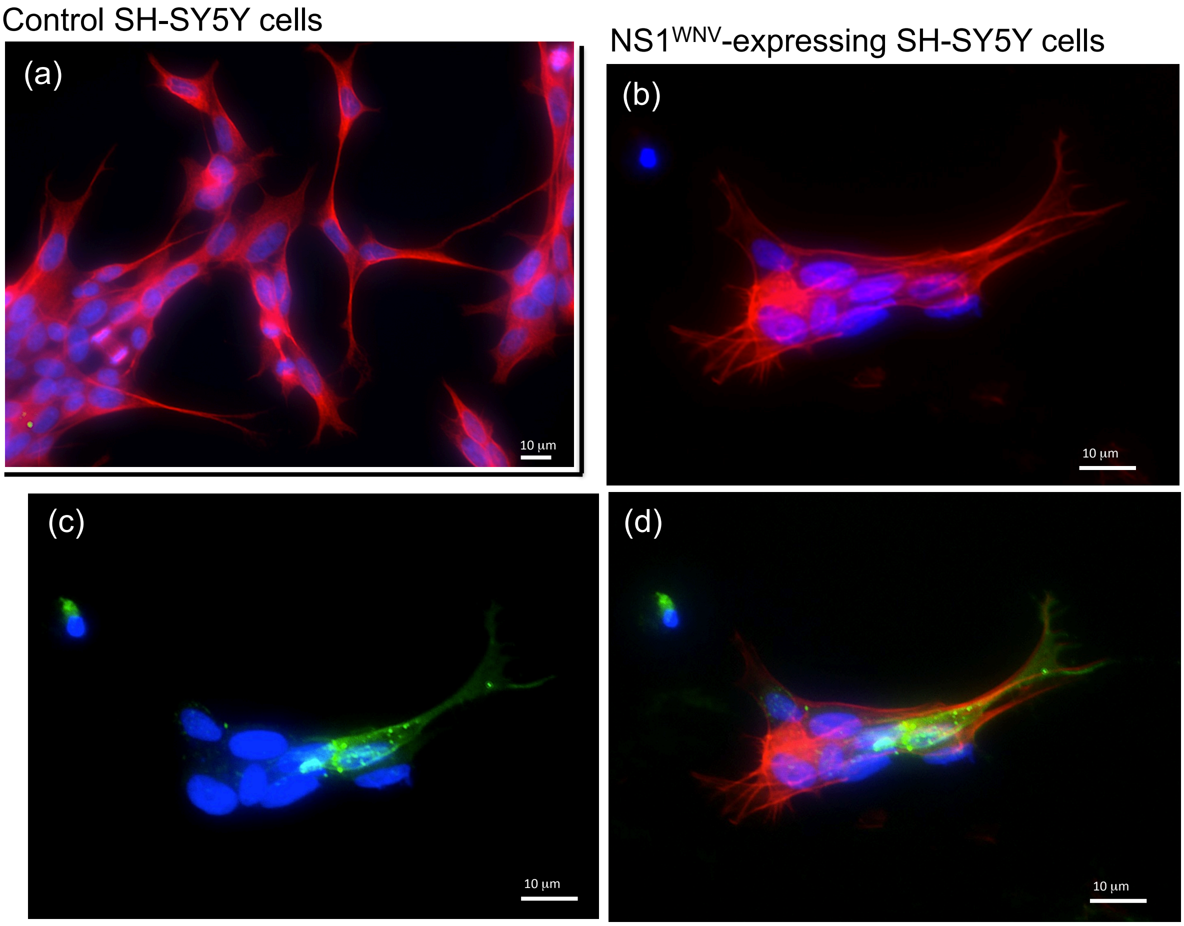

Supplementary Fig. S3. ***Actin network in recombinant NS1^{WNV}-expressing human neuronal cells SH-SY5Y.*** Cells cells were transfected with control empty plasmid pcDNA3.1 (**a**), or with pNS1$^{WNV\text{-}IS98}$ (**b-d**), and analyzed at 24h post-transfection by immunofluorescence confocal microscopy, using rhodamine-phalloidin for the labeling actin filaments, and specific rabbit antibody for the immunolabeling of NS1^{WNV}. Panels **(a,b)**, actin signal; **(c)**, NS1^{WNV} signal; **(d)**, merged signals. Nuclei were visualized in blue using DAPI staining. NS1^{WNV} signal is visible in actin-containing, cellular digitations.

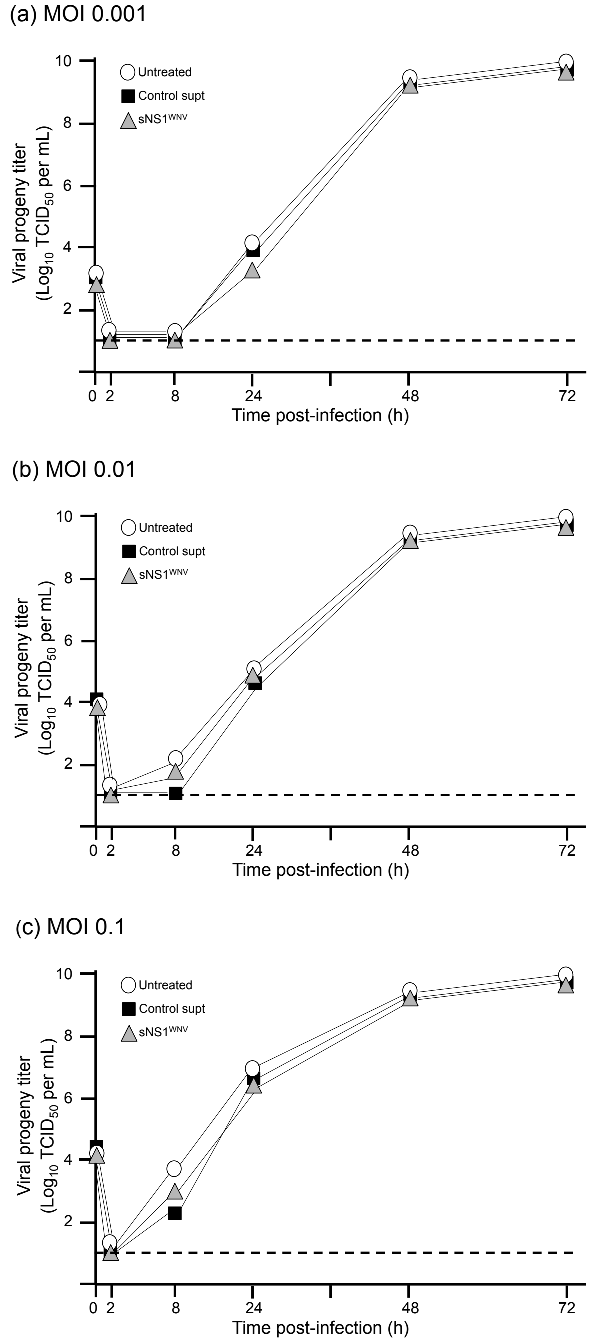

Supplementary Fig. S4. ***Viral growth in sNS1^{WNV}-treated Vero E6 cells.*** Aliquots of Vero E6 cells were preincubated with sNS1^{WNV} for 5hr at 37°C, before infection with WNV inoculum, at three different MOI, 0.001, 0.01 and 0.1, respectively. Control cultures consisted of untreated cells, and cells preincubated with samples from the cell culture medium of HEK-293T cells transfected with empty plasmid pcDNA3.1, and subjected to the same chromatographic process as the culture supernatant of pNS1$^{WNV\text{-}IS98}$-transfected HEK-293T cells. After rinsing with fresh DMEM, cells were further incubated at 37°C and 5% CO2 in DMEM supplemented with 10% FBS. Cell supernatants were collected at different times post-infection, and virus titers determined by end-point dilution assays in Vero E6 cells, using the Reed and Muench method. The results were expressed as $Log_{10}TCID_{50}$/mL. Data shown are mean of triplicate results from two separate experiments. The dotted line represents the limit of plaque detection with the method used. The same type of viral growth curves was obtained with the supernatants of Vero E6 cells incubated simultaneously with sNS1 and virus inoculum (data not shown).

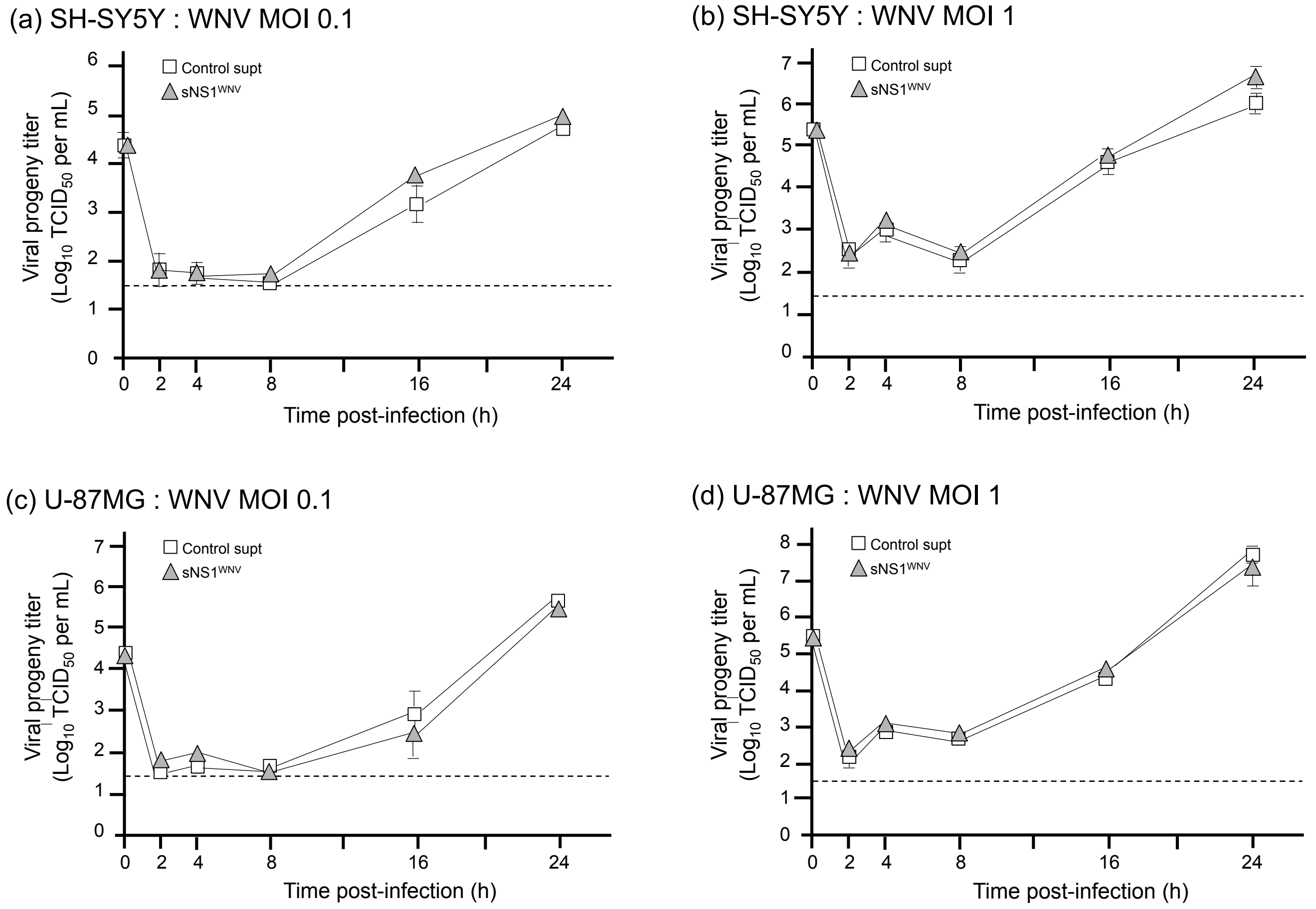

Supplementary Fig. S5. ***Viral growth in sNS1^{WNV}-treated SH-SY5Y and U-87MG cells.*** Aliquots of SH-SY5Y cells (**a, b**) or U-87MG cells (**c, d**) were preincubated with sNS1^{WNV} for 5hr at 37°C, before infection with WNV inoculum, at MOI 0.1 and 1, respectively. Control cultures consisted of untreated cells, and cells preincubated with samples from cell culture medium of HEK-293T cells transfected with empty plasmid pcDNA3.1, and subjected to the same chromatographic process as the culture supernatant of pNS1$^{WNV\text{-}IS98}$-transfected HEK-293T cells. After rinsing with fresh DMEM, cells were further incubated at 37°C and 5% CO2 in DMEM supplemented with 10% FBS for U-87MG, and in DMEM/F12 supplemented with 2% FBS for SH-SY5Y. Cell supernatants were collected at different times post-infection, and virus titers determined by end-point dilution assays in Vero E6 cells, using the Reed and Muench method. The results were expressed as $Log_{10}TCID_{50}$/mL. Data shown are mean of triplicate results from two separate experiments. The dotted line represents the limit of plaque detection with the method used. The same type of viral growth curves was obtained for the supernatants of SH-SY5Y and U-87MG cells incubated simultaneously with sNS1 and virus inoculum (data not shown).
